# Retrospective evaluation of autotransfusion using a cell saver device versus allotransfusion in the perioperative management of acute hemoperitoneum in 43 dogs (2017–2021)

**DOI:** 10.3389/fvets.2025.1465988

**Published:** 2025-02-04

**Authors:** Fabienne Blunschi, Dennis Gluding, Esther Hassdenteufel, Matthias Schneider, Hendrik Lehmann

**Affiliations:** Department of Veterinary Clinical Sciences, Small Animal Clinic, Justus-Liebig-University, Giessen, Germany

**Keywords:** blood salvage, autologous transfusion, spleen, hemangiosarcoma, blood transfusion, hemoabdomen

## Abstract

**Background:**

Cell saver (CS) technology is an increasingly popular approach for autotransfusion in small animal veterinary medicine for the treatment of patients with abdominal hemorrhagic effusion.

**Objective:**

To evaluate the utility, effectiveness, and safety of autotransfusions collected with a CS device and to assess whether the use of the CS device reduces the demand for allogenic blood transfusions.

**Materials and methods:**

Retrospective study of dogs with acute hemoperitoneum of splenic origin treated surgically. Dogs were grouped by the type of transfusion received: allo- and autotransfusion (AA), allotransfusion only (AO), autotransfusion only (CS), and no transfusion (NT). Differences in changes of laboratory parameters (hematocrit and lactate), transfusion volume, and outcomes were analyzed across groups.

**Results:**

Forty-three dogs were included. Twenty-seven (62.8%) suffered from hemangiosarcoma, and 16 (37.2%) had a benign cause of hemoperitoneum. The classification into blood transfusion groups was as follows: 7/43 (16.3%) in the AA-group, 11/43 (25.6%) in the AO-group, 11/43 (25.6%) in the CS-group and 14/43 (32.6%) in the NT-group. Increase in hematocrit over time was similar in all subgroups that received any form of blood transfusion (AA-, AO-, CS-group). Total volume of transfused blood (autologous and allogenic) was significantly higher in the AA-group (median 54.0mL/kg, range 24.7–126.5mL/kg) than in the AO-group (median 7.6mL/kg, range 4.6–13.5mL/kg, *p* = 0.01) but not the CS-group (median 23.8mL/kg, range 14.1–50.0mL/kg, *p* = 0.22). No difference was found for the volume of allogenic blood transfused between the AA-group (median 9.4mL/kg, range 5.0–16.2mL/kg) and AO-group (median 7.6mL/kg, range 4.6–13.5mL/kg) (*p* = 0.68). The use of the CS device did not adversely affect the time from presentation to surgery, the duration of surgery, or the outcomes.

**Discussion:**

The use of autologous blood transfusions obtained by CS device in dogs suffering from acute hemoperitoneum caused by a benign or malignant splenic disorder appeared safe and effective in the cases described. And therefore may emphasize its further application as an addition or alternative to traditional allogenic blood transfusions.

## 1 Introduction

Cell saver (CS) technology is an increasingly popular approach for the purpose of autotransfusion in small animal veterinary medicine as well as in human medicine for the treatment of patients with pleural or abdominal hemorrhagic effusion. Its use has been previously described in veterinary literature for dogs with intracavitary hemorrhage (1–4).

Several factors can explain the trend of favoring autotransfusions collected using a CS device over allotransfusions of stored blood. Maintaining a blood bank requires methodological expertise, specialized equipment like centrifuges and storage refrigerators, as well as the spatial capacity to accommodate this equipment. Furthermore, it is cost- and time-consuming and depends on a sufficient number of blood donors of the various blood groups (5). Insufficient amounts of stored blood products of the required blood group can limit treatment success in bleeding patients, which is especially true for emergency cases. Even with up-to-date standard blood banking, “storage lesions” occur, which could negatively affect survival of transfused red blood cells (RBC) and possibly result in adverse effects in recipients (6–8). In contrast, changes in morphology and function of RBCs are minor after cell salvage with CS (9). Although there is only a minor risk of transmitting infectious diseases from donor to recipient through blood transfusions (10, 11), this route of infection can be completely circumvented by the use of pure autologous blood. Even in cases of abdominal bacterial contamination, autotransfusion under application of a CS device may be suitable in the absence of alternatives as long as a leukocyte reduction filter is used, as this leads to an almost complete elimination of the bacterial burden of the recycled blood (12). Importantly, a recent veterinary study also showed that leukocyte reduction filters are effective in removing hemangiosarcoma (HSA) cells from the salvaged blood (13), making CS a reasonable approach to treat severe blood loss into body cavities from neoplastic diseases such as HSA. This is especially valuable since HSA is a frequent cause of acute hemoperitoneum in dogs (14).

Hemorrhagic disease of body cavities frequently requires aggressive intensive care measures, including blood transfusions to resuscitate the unstable cardiovascular system. Volume requirements can rapidly outnumber available stored blood products, making CS a valuable alternative. The applicability of CS in neoplastic disease is supported by human literature, which could not reveal an association between tumor recurrence or survival times and transfusion type (autotransfusion collected by CS vs. allotransfusion) (15). On the contrary, there is an ongoing controversy about a potential negative influence of allotransfusions on the long-term outcome of cancer patients due to its immunosuppressive effects on the recipient in human (16, 17) as well as in veterinary medicine (18). A potential risk of using the CS device is that large volumes of blood transfusion may increase the risk for adverse reactions. High-volume transfusions, such as massive transfusions, have been linked to various complications. These may include both acute and delayed transfusion reactions, hemolysis, hypothermia, hypocalcemia, hypomagnesemia, and organ dysfunction (19–22). However, hypocalcemia is not observed during autotransfusion, provided that the blood given does not use citrate as an anticoagulant (23, 24).

Lastly, but not least important, ethical concerns also need to be taken into consideration regarding allogenic blood products. Administering stored blood products for the treatment of patients with terminal disease (e.g., metastatic HSA) may exhaust blood storage and lead to insufficient available blood products for patients with a more favorable prognosis. Additionally, a potentially harmful blood donation must be performed in healthy donor dogs.

The objectives of this study were to evaluate the utility, effectiveness, and safety of autotransfusions collected with a CS device in dogs with hemoperitoneum of splenic origin and to assess whether the use of the CS device reduces the demand for allogenic blood transfusions. We hypothesized that dogs treated with the CS device would require fewer allogenic blood products and that there would be no difference in outcomes between the blood transfusion subgroups. Additionally, we hypothesized that there would be no difference in the change over time in HCT and lactate between the blood transfusion subgroups.

## 2 Materials and methods

### 2.1 Study design and inclusion criteria

The veterinary clinical database (easyVET, VetZ GmbH, Germany) of a university teaching hospital was searched for medical records of dogs that were presented with acute hemoperitoneum and associated surgical treatment between January 2017 and December 2021. The system was searched for the keywords “hemoperitoneum,” “hemoabdomen,” “cell saver,” and “hemangiosarcoma.” Inclusion criteria were complete data on signalment (breed, age, weight, sex), presence of hemoperitoneum of splenic origin only and performed splenectomy.

### 2.2 Diagnostics

Information regarding the medical history (clinical signs, presence of exercise intolerance, hypo/anorexia or dyspnea, event of collapse) and results of the general clinical examination were gathered. The time of the initial presentation was divided into emergency hours (18:00 to 07:30, as well as weekends and public holidays) and normal service hours (remaining time). Data on preoperatively (pre-OP) and postoperatively (post-OP) performed laboratory diagnostics were collected, including results of complete blood counts (CBC), biochemistry panels and venous blood gas analyses. Blood parameters of interest were hematocrit (HCT) (Procyte, IDEXX, Germany; Cobas b 123 blood gas analyzer, Roche, Germany; Cobas b 221 blood gas analyzer, Roche, Germany; ADVIA 2120, Siemens, Germany), blood lactate (Cobas b 123 blood gas analyzer, Roche, Germany; Cobas b 221 blood gas analyzer, Roche, Germany), ionized calcium (Cobas b 123 blood gas analyzer, Roche, Germany; Cobas b 221 blood gas analyzer, Roche, Germany; Stat Profile Prime, Nova Biomedical GmbH, Germany; Stat Profile ES Comp, Nova Biomedical GmbH, Germany) and ionized magnesium (Stat Profile Prime Nova Biomedical GmbH, Germany; Stat Profile ES Comp, Nova Biomedical GmbH, Germany). Dogs were classified as anemic based on the reference intervals of the hematological analyzing device used for HCT (Procyte ≤ 37.3%, blood gas analyzer ≤ 36.9%, ADVIA ≤ 39.0%). The course of blood lactate was assessed using lactate clearance calculated by the formula lactate clearance = lactate concentration ^pre-OP^ – lactate concentration ^post-OP^/lactate concentration ^pre-OP^× 100% (25). The change in HCT over time was evaluated using percentages. Trend data were used only if the different time-dependent blood parameter values were collected using the same laboratory analytical instruments pre- and post-OP. In patients with HCT measurements using both, hematology and blood gas analysis device, data obtained using the former were taken for analysis. If multiple measurements were performed in the pre- and/or post-OP periods, results of the first pre-OP measurement and of the measurement closest to the time point 12 h post-OP were chosen for the analysis. The 12-h timepoint was determined based on the average time between surgery and the next blood test and served to minimize the variance of this interval as far as possible.

For the assessment of potential electrolyte changes (hypocalcemia and hypomagnesemia) caused by the administration of blood products, the number of dogs with values below the reference range after receiving blood transfusions of any kind were counted.

For the evaluation of coagulation deviations, prothrombin time (PT) and activated partial thromboplastin time (aPTT) (STA Compact Max 3, Diagnostica Stago S.A.S., France; KC 4 A micro coagulometer, Heinrich Amelung GmbH, Germany) measurements were assessed and the time points of measurements (before or after surgery). Cases were searched for dogs with a prolongation of 1.5 times or higher the upper limit of the reference range (26).

Medical records were furthermore searched for the results of the pre-OP screening for metastatic disease. Assessed data included the type of diagnostic imaging (thoracic radiographs, abdominal ultrasound), estimation of the amount of free fluid (subjectively categorized as mild, moderate, or severe by the attending clinician using abdominal ultrasound), as well as presence and location of metastases. Abdominal ultrasound could either be a complete abdominal ultrasound or abdominal point-of-care ultrasound.

The histopathological diagnoses of the extirpated spleens were reviewed from the medical reports.

### 2.3 Treatment

Gathered surgical data included the duration from presentation to surgery, duration of surgery, technique of complete splenectomy (hemostasis using ligatures vs. vessel-sealing device) and occurrence of intraoperative complications (major bleeding or anesthetic incidents).

The type of blood products given [packed red blood cell (pRBC), fresh whole blood (FWB), fresh frozen plasma (FFP)] and their fractions, as well as volume (mL/kg) of allogenic and autologous blood were extracted from the medical records. For the calculation of given red blood cells (RBC) in milliliters, the full amount of pRBC transfusions and autotransfusions were used. In cases in which FWB was transfused, a conversion factor of 0.5 was applied to the volume administered to account for the lower packed cell volume in FWB (27). For the volume (mL/kg) of total RBCs (tRBC), transfused allogenic and/or autologous blood products were both taken into account and their respective volumes were added. If a dog received more than 90 mL/kg of blood (autologous or allogenic) in 24 h or more than half of the estimated blood volume within 3 hours, it was classified as a massive transfusion (19). If the information on the exact amount of milliliters of blood transfused was not recorded, the data of this dog were excluded from the calculation of the exact blood volume but were included for the description of the distribution of administrated blood type products. Plasma transfusions were evaluated as a separate value. FWB volume with a conversion factor of 0.5 was also added to the amount of plasma given to reflect the plasma content in FWB. Dogs only receiving plasma transfusions were assigned to the NT-group. It was further evaluated whether the administration of plasma was the consequence of coagulation testing (clinically relevant prolonged PT or aPTT). All allogenic blood products were collected, processed, and stored under the responsibility of an ACVECC board-certified veterinarian according to national blood banking guidelines (28). The same CS device (CellSaver 5+, Haemonetics GmbH, USA) was used for the collection of all autotransfusions. In all cases leukocyte reduction filters (RS Leukocyte removal Filter, Haemonetics GmbH, USA) were installed. In all instances, the CS device was utilized intraoperatively during open laparotomy. A standard midline approach through the linea alba was performed to gain access to the middle abdomen. Once access was achieved, the CS device was used to collect the hemorrhagic abdominal effusion, facilitating a better overview of the abdominal cavity for surgical exploration. A poole suction tip was employed to allow for a rapid evacuation of large volumes of blood. Complete removal of the abdominal hemorrhage was sought using the standard settings of the CS device. Autotransfusion was initiated right away after processing the blood, no additional anticoagulants were added to the washed blood. Depending on the timing of completion of processing, autotransfusions were administered either intra- or postoperatively.

### 2.4 Outcome

The duration of hospital stay, and outcome were analyzed. Medical records including anesthesia protocols and results of postoperative general physical examinations were searched for notes of complications related to the administered blood transfusions (e.g., hyperthermia, hypothermia, respiratory alterations, hypertension, signs of hemolysis). For evaluation of the outcome, telephone interviews with the owners were performed, in which it was ascertained whether their dogs were still alive at the time of the telephone call (February 2022). In cases of decease or euthanasia, date, and cause of death (related or unrelated to splenic or metastatic disease; unknown cause) were inquired. A relation was assumed if the patient developed similar clinical signs as preoperatively, but owners declined further diagnostics, or results of the diagnostic work-up were suggestive of metastases. Survival rates at discharge, 28-days, 6-months, and 1-year were explored.

### 2.5 Statistical analysis

Data sets were grouped according to the dignity of the pathological diagnosis (benign vs. malignant) and the origin of RBC-containing blood transfusion administered: allo- and autotransfusion (AA-group), allotransfusion only (AO-group), autotransfusion only (CS-group), and no transfusion (NT-group).

Statistical analyses and creation of graphs were performed using a statistical software program (IBM SPSS Statistics for Windows, version 26, IBM Germany GmbH, Germany). Continuous data were assessed for normality using visual evaluation of histograms and the Kolmogorov–Smirnov and Shapiro–Wilk tests. Data are presented as median and range. Normally distributed variables were compared using Student’s t-test, and non-normally distributed data were compared using the Mann–Whitney-U test. To determine the influence of one parameter on a second, non-dependent parameter, crosstab and chi-square n-1 test were used in two-by-two tables. When comparing values between more than two subgroups, Kruskal-Wallis test was used for non-normally distributed data and an ANOVA one-factor analysis of variance was used for normally distributed data. The used *post hoc* test was a Dunn’s test with Bonferroni correction to minimize the type I error with multiple comparisons. The Kaplan–Meier method was used to evaluate the survival characteristics of each subgroup. Statistical significance was set at *p* ≤ 0.05.

## 3 Results

The search revealed 69 results for the keywords “hemoperitoneum” or “hemoabdomen,” 50 for “cell saver,” and 95 for “hemangiosarcoma,” leading to a total of 214 cases. After removal of all duplicate cases, 180 cases (84.1%) remained. Of these remaining cases 49/180 (27.2%) were excluded because the dogs were either euthanized preoperatively or discharged for euthanasia at home without further treatment. Other reasons for exclusion were non-splenic origin of hemoperitoneum (46/180, 25.6%), hemoperitoneum due to complications of other surgeries (14/180, 7.8%), coagulopathies (10/180, 5.6%), hemangiosarcoma of the spleen without concurrent hemoperitoneum (9/180, 5.0%), CS usage for non-surgical procedures (e.g., blood washing for manual plasma exchange; 5/180, 2.8%) and inconclusive diagnoses (4/180, 2.2%). After excluding all these cases, 43/214 dogs (20.1%) were included for further analyses.

### 3.1 Signalment

Of the 43 dogs, 14 (32.6%) were intact males, 14 (32.6%) neutered males, 11 (25.5%) neutered females and 4 (9.3%) intact females. Breed affiliation was variable. Most commonly affected breeds were mixed breed dogs (*n* = 20, 46.5%), Labrador retriever (*n* = 5, 11.6%), and German Sheperd (*n* = 5, 11.6%). The median age at the time of presentation was 9.6 years (range 2.5–14.9 years). The median body weight of the presented dogs was 30.0 kg (range 8.8–56.0kg).

### 3.2 Clinical presentation

The most reported clinical sign was acute weakness (*n* = 39, 90.7%). Followed by tachycardia (*n* = 28, 68.3%) and pale (*n* = 16, 37.2%) to pale pink (*n* = 15, 34.9%) mucous membranes. The majority of patients were presented during emergency hours (*n* = 23, 53.5%). During emergency hours, 7/23 (30.4%) received an autotransfusion, whereas 11/20 (55.0%) of the patients that were seen during normal service hours were treated with the CS (*p* = 0.10).

### 3.3 Subgroups

Dignity of splenic disease: Of the 43 cases, 27 (62.8%) had a pathohistological diagnosis of splenic malignancy, of which all were HSA. The rest was diagnosed as benign origin (*n* = 16): hematoma (*n* = 7, 16.3%), nodular hyperplasia (*n* = 4, 9.3%), traumatic rupture of the spleen (*n* = 3, 7.0%), torsion (*n* = 1, 2.3%), and myelolipoma (*n* = 1, 2.3%).

Type of blood transfusion: Of all dogs, 7/43 (16.3%) received allogenic RBC transfusions in combination with an autotransfusion collected by CS (AA-group), 11/43 (25.6%) received allogenic RBC transfusions only (AO-group) and 11/43 (25.6%) received autologous blood only (CS-group). The remaining dogs (14/43, 32.6%) did not receive any kind of blood transfusion (NT-group). A total of 18/43 dogs (41.9%) received autologous blood (AA- and CS-group). A malignant origin of hemoperitoneum was determined in 3/7 (42.9%) in the AA-group, 9/11 (81.8%) in the AO-group, 8/11 (72.7%) in the CS-group, and 7/14 (50.0%) in the NT-group. The CS and AO-group had a higher percentage of dogs with malignant causes, but the difference failed to reach statistical significance (*p* = 0.23).

### 3.4 Diagnostic imaging

Thoracic radiographs were performed on 33/43 dogs (76.7%) and abdominal ultrasound was done in 42/43 cases (97.7%). One dog (2.3%) did not receive an ultrasound because it had already been done by the referring veterinarian. Of all 43 dogs, 5 (11.5%) had findings suggestive of the presence of metastases on presentation. In all five cases, metastases were visible on abdominal ultrasound. In one of these five cases, nodular lesions were additionally detected on thoracic radiographs suggesting metastatic lung disease. The estimates of the abdominal free fluid of the dogs of the respective subgroups are stated in Table 1. In three dogs (7.0%) a categorization of the quantity of hemoperitoneum was not noted in the medical records, and a retrospective categorization was not possible due to missing or insufficient ultrasound images.

**Table 1:** Volume of free abdominal fluid in dogs with acute hemoperitoneum of splenic origin classified as mild, moderate and severe and the subgroups of blood transfusion, allo- and autotransfusion (AA-group), allotransfusion only (AO-group), autotransfusion only (CS-group) and no transfusion at all (NT-group).

Classification	Mild *n* (%)	Moderate *n* (%)	Severe *n* (%)	Not classified *n* (%)
Subgroup				
AA-group	0 (0%)	4 (57.1%)	1 (14.3%)	2 (28.6%)
AO-group	1 (9.1%)	6 (54.6%)	3 (27.3%)	1 (9.1%)
CS-group	0 (0%)	8 (72.7%)	3 (27.3%)	0 (0%)
NT-group	7 (50.0%)	6 (42.8%)	1 (7.1%)	0 (0%)

### 3.5 Surgical procedure

In 32/43 cases (74.4%), records were available for evaluation of the time period from presentation until start of surgery. The overall median time was 147 min (min) (range 60–1,260 min). There was no difference in this length of time regarding the different blood transfusion subgroups (*p* = 0.90). Regarding the blood transfusion subgroups, the median time was 110 min (range 75–1,260 min) in the AA-group, 140 min (range 105–390 min) in the AO-group, 160 min (range 60–720 min) in the CS-group, and 132.5 min (range 65–470 min) in the NT-group. Complete splenectomy was performed in all cases. In the majority (*n* = 40, 93.0%), hemostasis was performed using a vessel sealing device (LigaSure, Medtronic, Germany). The applied technique was not recorded in the remaining three cases (7.0%). Information regarding the duration of surgery was available in 28/43 cases (65.1%). Median duration was 87.5 min (range 35–160 min). There was no significant difference in median duration between the AA-group (60.0 min, range 60–90 min), AO-group (97.5 min, range 35–150 min), the CS-group (90.0 min, range 45–160 min), and the NT-group (90.0 min, range 45–140) (*p* = 0.45). No surgical intraoperative complications were reported in the medical records.

### 3.6 Blood transfusion

#### 3.6.1 Type of allogenic blood transfusions

In the AA-group 5/7 dogs (71.4%) received pRBC and 2/7 dogs (28.6%) were given a FWB transfusion. None of the dogs received both. In the AO-group, 6/11 dogs (54.5%) received pRBC only and 4/11 dogs (36.4%) FWB only, while 1/11 (9.1%) received both pRBC and FWB. Of the dogs receiving pRBC, 1/6 (16.7%) received two units of pRBC and the other 5/6 (83.3%) were given only one unit.

##### 3.6.1.1 Plasma

A total of 18/43 dogs (41.9%) received a plasma containing transfusion (FFP or FWB). In the AA-group, 7/7 dogs (100%) received a plasma transfusion, of which 5/7 (71.4%) were given FFP only, 1/7 (14.3%) FWB only and 1/7 (14.3%) FFP and FWB. In the AO-group, 7/11dogs (63.6%) received plasma. Of these, 2/7 (28.6%) received FFP only, 3/7 (42.8%) FWB only and 2/7 (28.6%) both FFP and FWB. In the CS-group, 4/11 dogs (36.4%) were transfused with plasma, of which all four dogs received FFP only. None of the dogs in the NT-group received plasma. When looking at whether the dogs needed plasma after autotransfusion 11/18 (61.1%) dogs with autotransfusion received plasma while 7/25(28%) dogs not receiving autotransfusion were transfused with FFP. The incidence between the two groups (autologous blood yes or no) was not significant (*p* = 0.06).

Plasma was given in 10/18 dogs (55.6%) after coagulation testing was conducted, which revealed hypocoagulability. In dogs without coagulation testing performed, 2/8 cases (25%) received plasma in the form of FWB transfusion in order to treat a suggested coagulation disorder noticed by diffuse bleeding from manipulated serosal surfaces. In the remaining 6/8 cases (75%), it was given at the discretion of the attending veterinarian without any recorded reason. Regarding the timing of plasma administration, it was given to 7/18 dogs (38.9%) prior to surgery, to 1/18 (5.6%) during surgery and to 6/18 (33.3%) dogs after surgery. Additionally, two dogs (11.1%) received plasma both before and after surgery, while for two dogs (11.1%), it could not be determined whether plasma was given before or after surgery.

#### 3.6.2 Volume of blood transfusion

##### 3.6.2.1 Allogenic RBC transfusion

Data concerning the volume of allogenic RBC was available for 17/18 dogs (94.4%). The median volume of allogenic RBC transfused was 8.7mL/kg (range 4.6–16.2mL/kg). Analyzed by subgroup, no difference was found between the AA-group (median 9.4mL/kg, range 5.0–16.2mL/kg, *n* = 7) and the AO-group (median 7.6mL/kg, range 4.6–13.5mL/kg, *n* = 10) (*p* = 0.68). Additionally, dignity subgroups did not possess a significant difference (*p* = 0.10): benign group (median 10.0mL/kg, range 5.2–16.2mL/kg, *n* = 6) and malignant group (median 7.5mL/kg, range 4.6–12.2mL/kg *n* = 11).

##### 3.6.2.2 Autotransfusion

Evaluation of the transfused volume of autotransfusion was possible for 14/18 dogs (77.8%). The median volume of all dogs (AA and CS-group) was 33.3 mL/kg (range 14.0–117.1 mL/kg, *n* = 14). Missing data did not allow assessment for two cases of each group. In the AA- and the CS-group, the median volumes of autologous blood transfused were 44.0 mL/kg (range 19.4–117.1 mL/kg, *n* = 5) and 23.8 mL/kg (range 14.1–50.0 mL/kg, *n* = 9) respectively (*p* = 0.24). Dogs of the benign group were given a median volume of 31.3 mL/kg (range 19.4–117.1mL/kg, *n* = 5) and dogs belonging to the malignant group were treated with a median volume of 23.8mL/kg (range 14.1–53.5 mL/kg, *n* = 9) (*p* = 0.39).

##### 3.6.2.3 Total RBC transfusion

**Figure 1:**
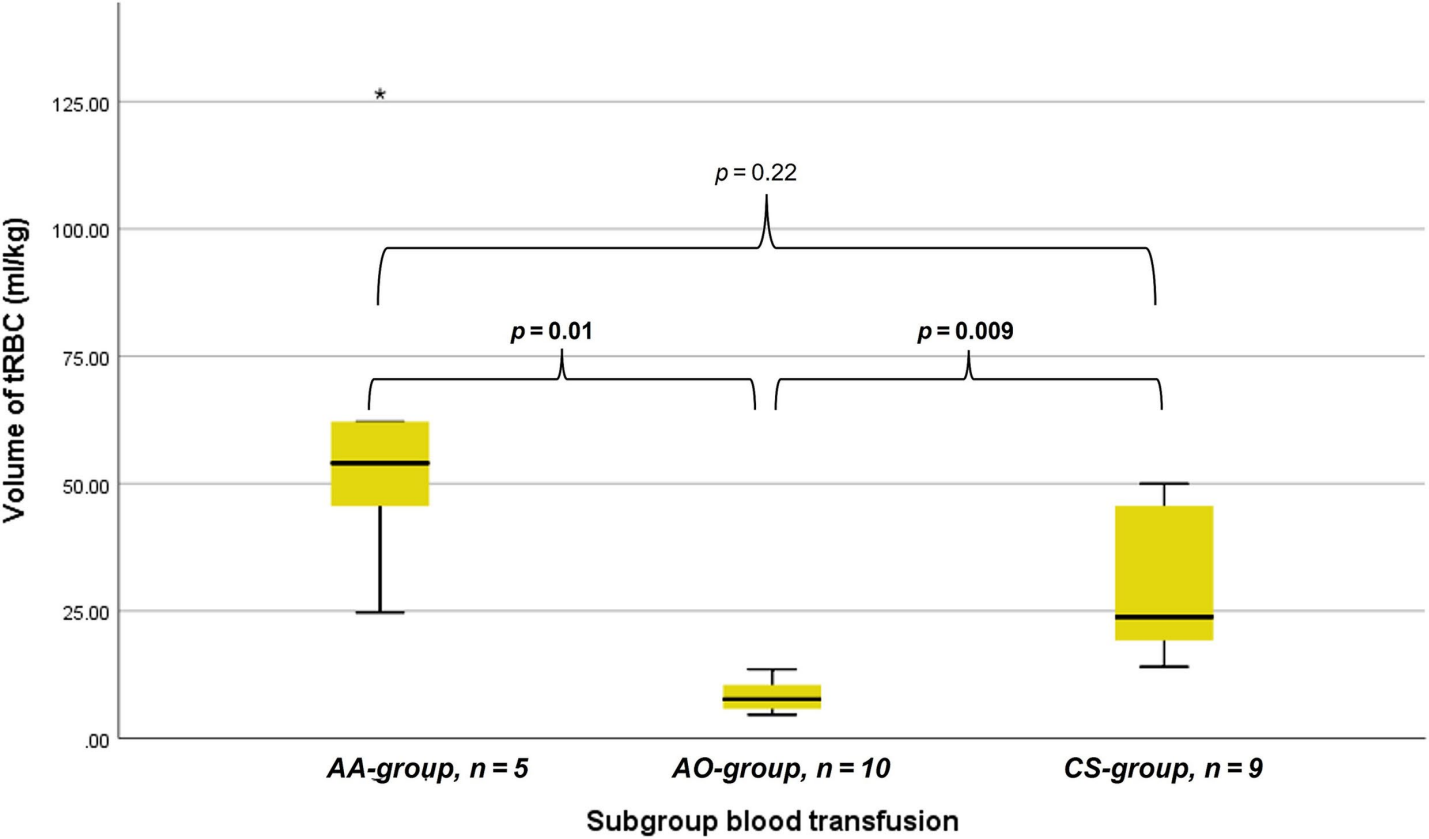
Box whisker plots depicting the transfused volume of total red blood cell containing blood (tRBC) in milliliters per kilogram bodyweight considering dogs that received allo- and autologous blood (AA) allogenic blood only (AO), and autologous blood only with cell saver (CS). *p*-values are provided above the horizontal brackets, with significant *p*-values in bold.

The tRBC volume could be calculated for 5/7 dogs (71.4%) in the AA group. These five received a median of 54.0 mL/kg (range 24.7–126.5 mL/kg) tRBC, which is significantly higher than the transfused volumes of tRBC in the AO-group (median 7.6mL/kg; range 4.6–13.5 mL/kg, *n* = 10) (*p* = 0.01) but not the CS-group (median 23.8 mL/kg, range 14.1 – 50.0 mL/kg, *n* = 9) (*p* = 0.22). Furthermore, the CS-group did receive a higher volume of tRBC per kilogram than the AO-group (*p* = 0.009). The amount of tRBC given per subgroup is shown in Figure 1. Dogs in the benign group received a median amount of tRBC of 31.3 mL/kg (range 10.5–126.5mL/kg, *n* = 7) and dogs in the malignant group 14.1 mL/kg (range 4.6–62.2 mL/kg, *n* = 17) (*p* = 0.11). A total of seven dogs received amounts classified as massive transfusion. Of these, six received more than 45 mL/kg in 3 hours and one by receiving a total amount of more than 90 mL/kg in 24 h. Of these seven dogs, four were in the AA-group and three were in the CS-group.

##### 3.6.2.4 Allogenic plasma

For the plasma volumes transfused, data was available for all 18 dogs that either received a FFP or FWB transfusion. The overall median amount was 10.7 mL/kg (range 4.6 to 23.2mL/kg, *n* = 18). The difference between the AA-group (median 19.9 mL/kg, range 7.8 to 22.2mL/kg, *n* = 7), the AO-group (median 8.5mL/kg; range 4.6 to 13.0mL/kg, *n* = 7) and the CS-group (median 12.5, range 10.0–23.2mL/kg, *n* = 4) failed to reach statistical significance (*p* = 0.08).

### 3.7 Laboratory parameters

Information regarding pre-OP and post-OP HCT was available for 33/43 dogs (76.7%). At the time of presentation, 25/33 dogs (75.8%) were anemic. Dogs in the malignant group were more often anemic pre-OP (*n* = 18/20, 90%) than in the benign group (*n* = 7/13, 53.9%) (*p* = 0.02). The frequency of a pre-OP anemic condition regarding subgroups was reported as follows: In the AA-group 6/7 (85.7%), in the AO-group 8/8 (100%), in the CS-group 7/9 (77.8%) and in the NT-group 4/9 (44.4%). The median pre-OP HCT of the benign group was 37.0% (range 23.0–50.2%, *n* = 13) and therefore significantly higher than the median HCT of the malignant group, 29.2% (range 13.9–41.0%, *n* = 20) (*p* = 0.01). Median HCT pre-OP (*p* = 0.26) and post-OP (*p* = 0.45) were not different across blood transfusion subgroups. Values are depicted in Table 2. The percentage change in HCT was not significantly different between groups (*p* = 0.35). The AA-group showed a median change of −14.3% (range − 32.1 – 160.7%), the AO-group of +0.9% (range − 24.3 – 32.7%), the CS-group of +2.1% (range − 25.9 – 151.8%) and the NT-group of −7.7% (range − 59.0 – 2.4%).

**Table 2:** Pre- and postoperative hematocrit of dogs with surgically treated acute hemoperitoneum of splenic origin between the subgroups of blood transfusion received, allo- and autotransfusion (AA-group), allotransfusion only (AO-group), autotransfusion only (CS-group) and no transfusion at all (NT-group) as well as dignity of the underlying cause, benign and malignant.

	Pre-OP			Post-OP	
Subgroup	*n*	Median (%)	Range (%)	Median (%)	Range (%)
AA-group	7	28.6	21.2–47.0	28.8	19.0–55.0
AO-group	8	28.8	15.8–38.0	26.0	19.6–34.0
CS-group	9	29.4	13.9–47.0	33.0	20.0–48.0
NT-group	9	36.6	23.5–50.2	29.7	19.9–44.0
Benign group	13	37.0	23.0–50.2	25.8	19.0–48.0
Malignant group	20	29.2	13.9–41.0	31.0	19.6–55.0

Auto- and allotransfusion (AA), allotransfusion only (AO), autotransfusion via cell saver (CS), no transfusion (NT), number (*n*), preoperative (pre-OP), postoperative (post-OP).

Lactate concentrations pre-OP and post-OP are summarized in Table 3. Data was available for 22/43 dogs (51.2%). There was a difference in the pre-OP lactate concentrations regarding the subgroups of blood transfusions (*p* = 0.03). The AA-group showed higher median concentrations compared to the NT-group (*p* = 0.04) but not the AO-group (*p* = 0.2) and CS-group (*p* = 0.5). Such a difference could no longer be detected in the post-OP values (*p* = 0.40). There was no difference between the benign group and the malignant group in the pre-OP (*p* = 0.63) and post-OP values (*p* = 0.25). The post-OP lactate concentrations were lower in all groups when compared to the respective pre-OP values (Table 3). When lactate clearance was considered, there was no difference between the subgroups of blood transfusion (*p* = 0.10). Median lactate clearance was 79% (range 58 94%, *n* = 3) in the AA-group, 70% (range 45 – 85%, *n* =  6) in the AO-group, 69% (range 31 – 88%, *n* = 7) in the CS-group and 48% (range − 150  – 79%, *n* = 6) in the NT-group.

**Table 3:** Pre- and postoperative blood lactate concentration of dogs with surgically treated acute hemoperitoneum of splenic origin between the subgroups of blood transfusion received, allo- and autotransfusion (AA-group), allotransfusion only (AO-group), autotransfusion only (CS-group) and no transfusion at all (NT-group) as well as dignity of the underlying cause, benign and malignant.

		Pre-OP		Post-OP	
Subgroup	*n*	Median (mmol/L)	Range (mmol/L)	Median (mmol/L)	Range (mmol/L)
AA-group	3	12.2	9.8–13.3	2.0	0.8–5.1
AO-group	6	5.8	4.0–8.4	1.6	1.3–3.2
CS-group	7	7.5	2.9–13.2	1.7	1.0–2.6
NT-group	6	4.4	1.5–9.5	2.5	1.3–7.8
Benign group	7	7.2	2.9–9.8	2.6	1.0–7.8
Malignant group	15	5.9	1.5–13.3	1.7	0.8–5.1

Auto- and allotransfusion (AA), allotransfusion only (AO), autotransfusion via cell saver (CS), no transfusion (NT), number (n), preoperative (pre-OP), postoperative (post-OP).

Ionized calcium was measured in 26/29 patients (89.7%) post blood transfusion (AA-, AO-, CS-group). Of these dogs 5/26 (19.2%) were hypocalcemic (range 1.04–1.20 mmol/L), but none of the dogs had to be treated for clinical hypocalcemia. Of these five dogs with hypocalcemia, two (40.0%) belonged to the AA-group, one (20.0%) to the AO-group and two (40.0%) to the CS-group. Among the seven dogs that underwent massive transfusion, two were found to be hypocalcemic afterwards, with the lowest recorded ionized calcium concentration at 1.10 mmol/L. Again, no calcium substitution was performed due to the absence of clinical signs.

Ionized magnesium post blood transfusion was measured in 18/29 dogs (62.1%). Of these 12/18 (66.7%) had magnesium concentrations within the reference range of the respective devices used for measurement. Low magnesium concentrations were measured in 5/18 dogs (27.8%) with a range of 0.33–0.46 mmol/L. One dog had a magnesium concentration measured above the reference range (0.66 mmol/L). Of these five dogs with low magnesium concentrations, two (40.0%) belonged to the AA-group, one (20.0%) to the AO-group and two (40%) to the CS-group. Out of the seven dogs that received massive transfusion, ionized magnesium concentrations were recorded for five of them. One dog was found to be hypomagnesemic post-transfusion, with a measured ionized magnesium concentration of 0.33 mmol/L, while the other four dogs had ionized magnesium concentrations within the normal reference range.

For a total of 17 dogs, PT and aPTT testing was performed prior to surgery. Of these, PT was prolonged in 14/17 cases (82.4%) and aPTT in 9/17 cases (52.9%). Two dogs with prolonged PT had a PT prolongation of more than 1.5 times the upper limit. Additionally, seven dogs, including the former two dogs, had an aPTT prolongation of more than 1.5 times the upper limit. After surgery, 16 dogs had PT and aPTT measurements performed, PT was prolonged in 13/16 dogs (81.3%) of which two dogs had a prolongation of more than 1.5 times the upper limit. Both dogs belonged to the AA-group. One of these two dogs did not have coagulation testing done before surgery and the second dog had a prolongation of PT prior to surgery and autotransfusion, but less than 1.5 times the upper limit. Twelve of the 16 dogs (75%) had a prolonged aPTT, of which five dogs had a prolongation of more than 1.5 times the upper limit, four of these belonged to the AA-group and one to the CS-group. Of these five dogs with more than 1.5 times aPTT prolongation, three did not have coagulation testing before surgery and the other two had prolonged aPTT (less than 1.5 times prolongation).

### 3.8 Outcome

The median duration of hospital stay was 3 days (d) (range 0–13d, *n* = 43). There was neither a difference between the benign group (median 3d, range 0–9d, *n* = 16) and the malignant group (3d, range 0–13d, *n* = 27) (*p* = 0.78) nor between the subgroups of blood transfusion: AA-group (5d, range 0–13d, *n* = 7), AO-group (3d, range 1–7d, *n* = 11), CS-group (3d, range 1–9d, *n* = 11), and NT-group (2d, range 0–4d, *n* = 14) (*p* = 0.16). Documented data regarding implemented transfusion reaction monitoring was sparse, but no evidence was found within the medical records for complications directly linked to the performed transfusions. Thirty-nine of the 43 dogs (90.7%) survived until discharge. Of the four dogs that did not survive, one was in the AA-group, two were in the CS-group and one in the NT-group. The in-house mortality rate for dogs receiving autologous blood transfusion was 16.7 and 4% for dogs receiving no autologous blood. Of the four deceased dogs, the dog of the NT-group was euthanized due to clinical and laboratory deterioration (tachycardia, weakness, anemia, hypalbuminemia) on behalf of the owners, refusing blood transfusions due to financial constraints. The dog in the AA-group that did not survive to discharge suffered a cardiovascular arrest during induction of general anesthesia, after successful resuscitation, splenectomy was performed. Following the successful resuscitation, the dog exhibited systemic hypotension that was unresponsive to fluid therapy and demonstrated only a limited reaction to the initiated vasopressor treatment. Furthermore, the dog progressively developed cardiac arrhythmias and showed pulseless ventricular tachycardia, which triggered a second cardiac arrest in the postoperative phase that was again successfully resuscitated. Afterwards, the patient showed severe neurological deficits without autonomous respiratory activity. Electroencephalography showed no detectable brain function and euthanasia was elected. One of the dogs in the CS-group was euthanized due to severe aspiration pneumonia and sepsis and the other died due to cardiopulmonary arrest 6 h after surgery. The underlying reason for this could not be determined. Resuscitation was rejected by the owner. Only one dog not surviving until discharge received a massive transfusion.

The follow-up interview was performed after a median time interval of 554 d after the surgical procedure (range 5–1919 d). Thirty-seven of 39 owners (94.8%) of discharged dogs responded.

Of these dogs, 29/39 (74.3%) were alive after 28 days, 15/39 (38.4%) after 6 months and 12/39 (30.7%) after 1 year. The survival between all subgroups is shown in Table 4. In the benign group, 8/16 (50.0%) had passed away or were euthanized at the time of the interview (median 867 d, range 163–1,589 d after surgery), 7/16 (43.8%) were alive and for 2/16 (12.6%) no information could be gathered. In the malignant group, 24/27 dogs (88.9%) were deceased or euthanized at the time of the interview (median 418 d, range 5–1919d after surgery), survival was reported for 2/27 dogs (7.4%), and for 1/27 (3.7%) follow-up data was missing.

**Table 4:** Absolute and relative numbers of dogs alive at hospital discharge as well as 28 days, 6 months and 1 year after hospital discharge listed by subgroups of blood transfusion received, allo- and autotransfusion (AA-group), allotransfusion only (AO-group), autotransfusion only (CS-group) and no transfusion at all (NT-group) as well as dignity of the underlying cause, benign and malignant.

	Discharge *n* (%)	28 days *n* (%)	6 months *n* (%)	1 year *n* (%)
AA-group (*n* = 7)	6 (85.7%)	4 (57.1%)	3 (42.9.4%)	2 (28.6%)
AO-group (*n* = 11)	11 (100%)	6 (54.55%)	2 (18.2%)	2 (18.2%)
CS-group (*n* = 11)	9 (81.8%)	7 (63.6%)	3 (27.3%)	3 (27.3%)
NT-group (*n* = 14)	13 (92.9%)	12 (85.7%)	7 (50.0%)	5 (35.7%)
Benign group (*n* = 16)	14 (87.5%)	12 (75.0%)	12 (75.0%)	10 (62.5%)
Malignant group (*n* = 27)	25 (92.6%)	17 (63.0%)	3 (11.1%)	2 (7.4%)

Auto- and allotransfusion (AA), allotransfusion (AO), autotransfusion (CS), no transfusion (NT), number (*n*).

**Figure 2:**
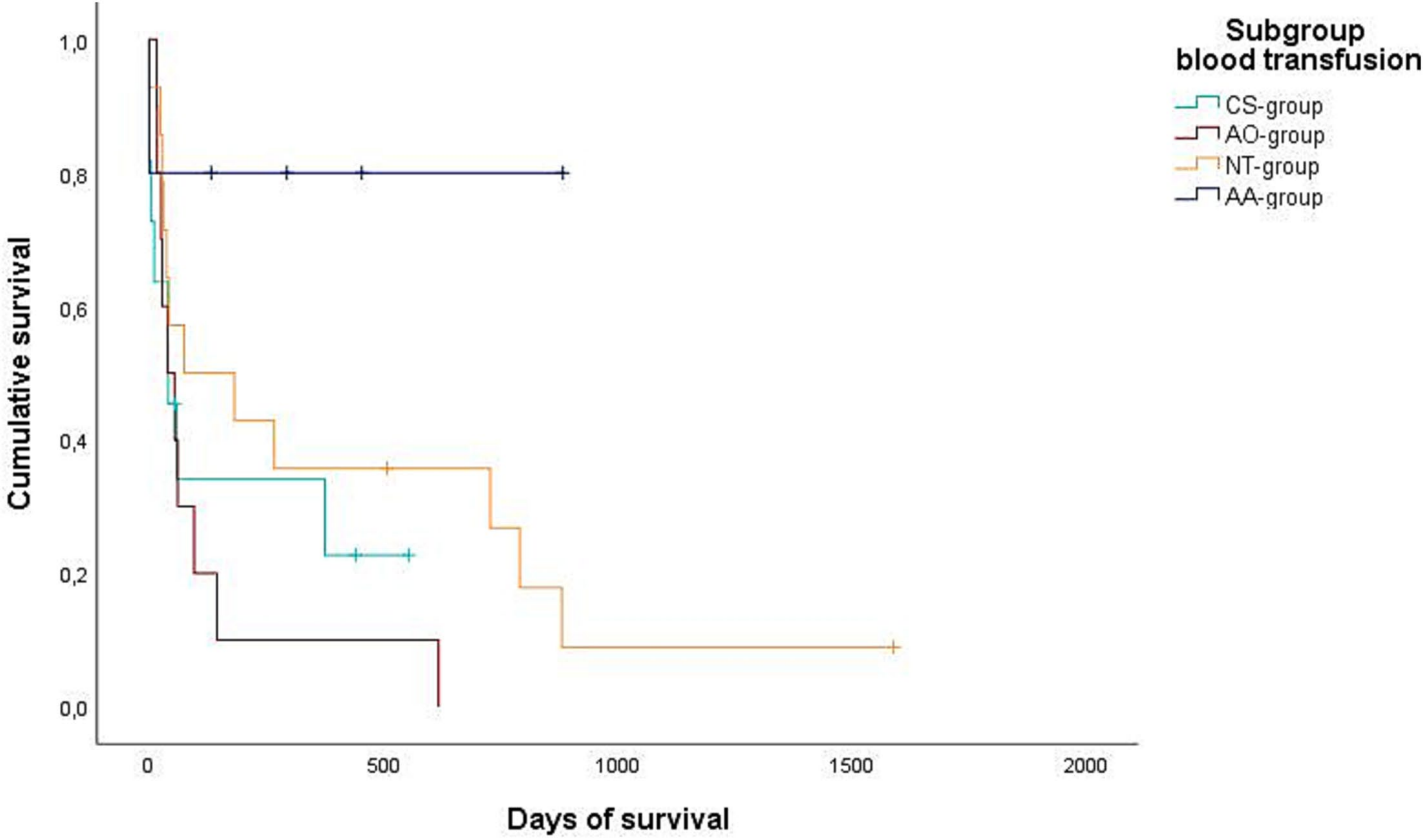
Kaplan–Meier plot showing the postoperative cumulative survival in days of all dogs considering the different blood transfusion subgroups [combined auto- and allotransfusion, *n* = 5 (AA), allotransfusion only, *n* = 10 (AO), autotransfusion collected via cell saver *n* = 11 (CS), and no transfusion, *n* = 14 (NT)].

**Figure 3:**
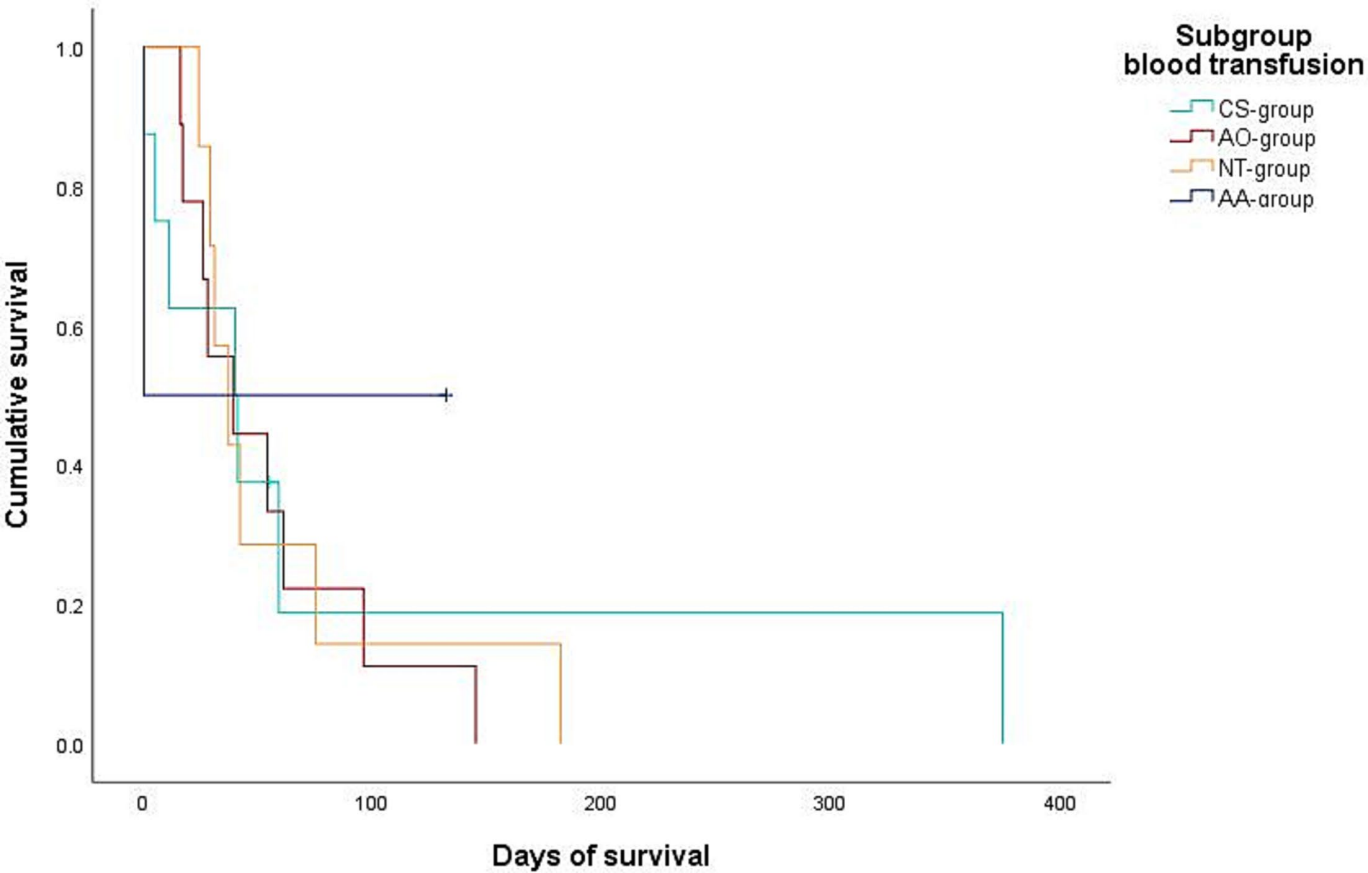
Kaplan–Meier plot showing the postoperative cumulative survival in days of dogs with diagnosed splenic malignancy (malignant-group) considering the different blood transfusion subgroups [combined auto- and allotransfusion, *n* = 5 (AA), allotransfusion only, *n* = 9 (AO), autotransfusion collected via cell saver, *n* = 5 (CS), and no transfusion, *n* = 7 (NT)].

**Figure 4:**
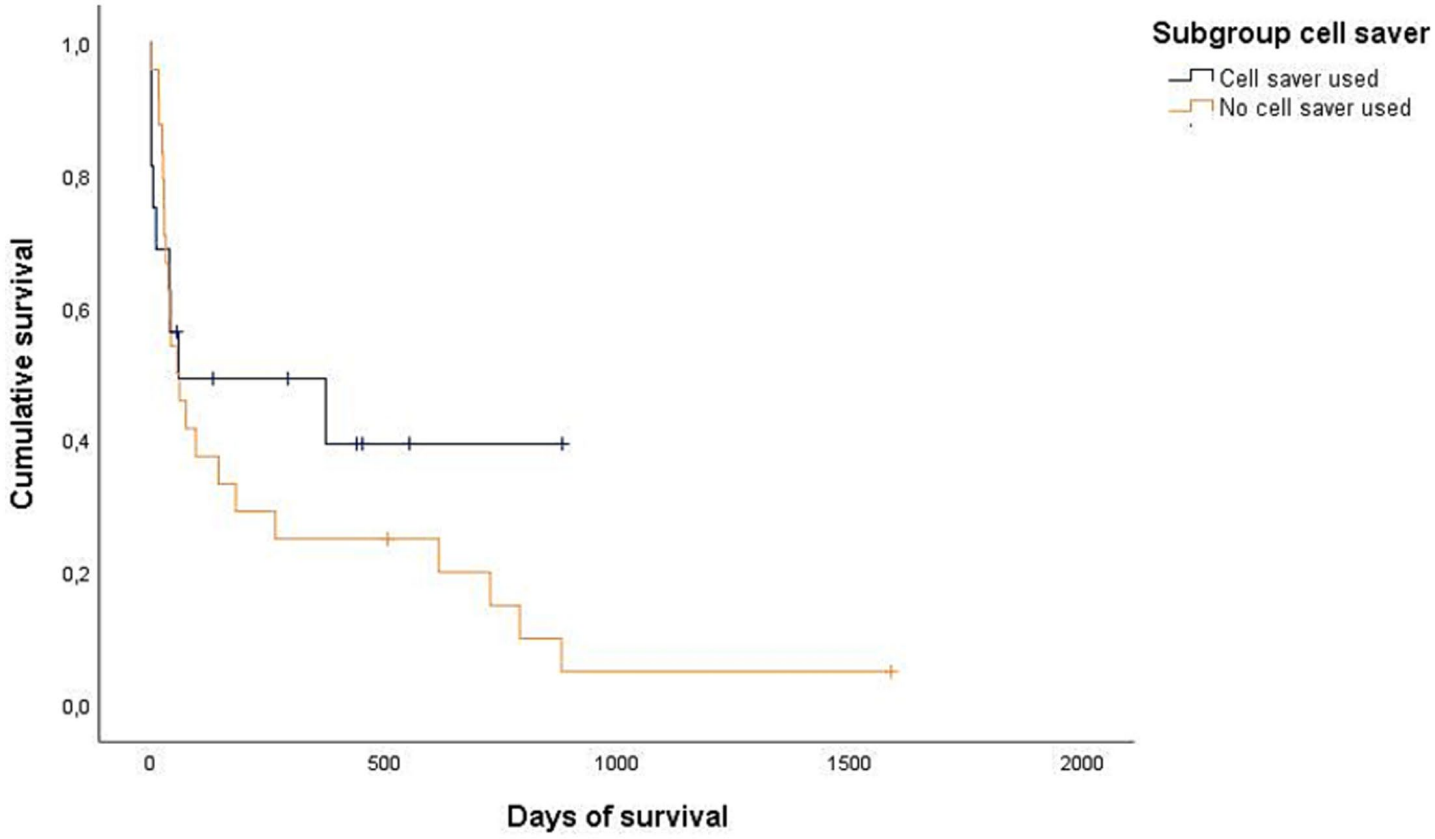
Kaplan–Meier plot showing the postoperative cumulative survival in days of all dogs considering whether a cell saver device was used (*n* = 16) or not (*n* = 24).

**Figure 5:**
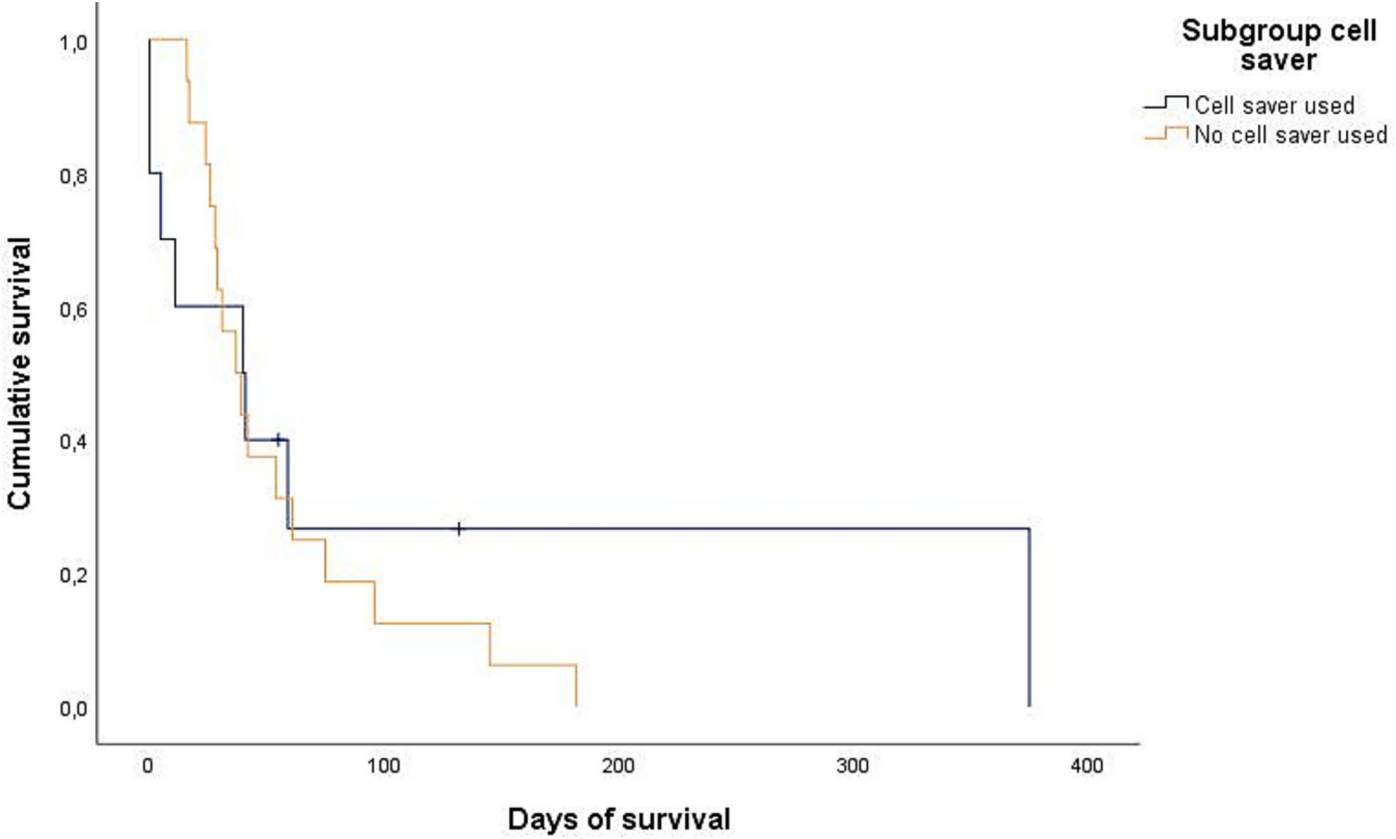
Kaplan–Meier plot showing the postoperative cumulative survival in days of dogs with diagnosed splenic malignancy (malignant-group) considering whether a cell saver device was used (*n* = 10) or not (*n* = 16).

Data regarding the cause of death were obtained for 29/32 dogs (90.6%) either by medical records or by telephone interview. In 25/29 of these dogs (86.2%), medical history, clinical examination in combination with further diagnostics such as histopathology, diagnostic imaging and/or laboratory analyses revealed a certain, or at least a highly likely, relation, between the cause of death and splenic or metastatic disease. Of these 25 dogs, 2/25 (8%) belonged to the benign group and 23/25 (92%) belonged to the malignant group. All 23 dogs of the malignant group died or were euthanized due to the malignancy and its associated hemoperitoneum or its metastatic disease. Both dogs with benign causes and associated deaths were either euthanized or died during hospital stay. Kaplan–Meier curves depict the survival subdivided according to blood transfusion subgroups (Figures 2, 3) and whether a CS-device was used or not (Figures 4, 5). Due to the infrequent associated deaths in dogs of the benign group, in addition to the Kaplan–Meier curves of all dogs (Figures 2, 4), they were repeated with only the malignant group included (Figures 3, 5).

## 4 Discussion

In this retrospective study of 43 dogs with hemoperitoneum of splenic origin, data illustrate that the use of autologous blood obtained by a CS device can serve as an appropriate way to treat internal blood loss without having a negative impact on time from presentation to surgery, duration of surgery, outcome of surgery or survival times compared to dogs receiving allogenic blood products. The in-hospital mortality rate was low in all subgroups, which is why no statement can be made regarding a possible difference. This result is consistent with previous publications about autotransfusion using a CS (2, 3) and may encourage the use of CS in patients with hemoperitoneum of splenic origin, even in cases when HSA is suspected.

All variants of RBC transfusions (allogenic and autologous) proved useful to stabilize or increase the postoperative HCT. Accordingly, transfusion of autologous blood processed by a CS device may be regarded as a reliable method for a clinically valuable stabilization of HCT. Although the study results reveal that the availability of a CS device cannot render the storage of blood products unnecessary, as some dogs treated with the CS device needed additional allogenic blood products, it can be emphasized that eleven dogs (CS-group) were managed solely with an administration of autologous blood alone with similar results. It is likely that the treatment of some of those dogs might also have required allogenic blood products without the application of the CS device, based on their severe clinical hemodynamic parameters at the time of presentation. The assumption that dogs in the CS-group would have required an allogenic blood transfusion without the use of the CS device is also supported by the fact that the amount of abdominal free fluid detected by ultrasound was estimated as moderate or severe in all these dogs, comparable with the dogs of the AA- and AO-group. In comparison, dogs that had not received a transfusion had only a mild amount of free fluids in 50% of cases. Considering the similar amount of allogenic blood used in the AA-group and the AO-group, the question arises as to whether there are preoperative factors that may predict the likelihood of the necessity of additional allogenic blood products. A direct correlation of blood lactate concentrations and the severity of clinical symptoms has not been described for hemoperitoneum of splenic origin in dogs, but numerous previous studies investigating other diseases (e.g., gastric dilation and volvulus, immune mediated hemolytic anemia, babesiosis) (29–31) and critically ill dogs (25, 32) indicate that a higher lactate concentration is indicative of a more pronounced hemodynamic instability, although a clear consensus on its clinical applicability has not yet been reached (33). A study in human medicine that examined lactate as an indicator of blood requirement also found that higher lactate concentrations can be used as an indicator for the requirement of large volume blood transfusion (34). Considering this, the higher initial lactate concentrations in the AA-group may indicate a worse clinical condition at the time of presentation compared to the other dogs and therefore higher transfusion requirements, which may explain the higher volume of tRBC needed. Due to the retrospective design of the study, it was not possible to determine whether the dogs in the AA-group received allogenic blood before surgery or during surgery, alongside the autologous blood, as the autologous blood alone was insufficient to improve their clinical status. It is possible that dogs in the AA-group would not have required the full amount of tRBC, or that the amount of autologous blood transfusion would have been sufficient, but the administration of allogenic blood was given prior to the use of autologous blood transfusion for initial stabilization. To avoid this factor, a prospective randomized study would be necessary. Nevertheless, a relation between high preoperative blood lactate concentrations and the need for a higher amount of blood products appears plausible. The higher total volume of administered blood with autotransfusion (CS-group) compared to the allogenic transfusion only (AO-group) may be explained by the circumstances, that, in part the volume of allogenic blood transfusions depends on the volume of the available blood stored at the time of surgery as well as summation of cost per blood unit, while the quantity in the CS-group depends mostly on the amount of blood in the abdomen. Therefore, a more restrictive administration may be chosen for allogenic blood products due to those cost and stock limitations.

The analysis of the usage distribution of the CS device revealed a tendency for increased utilization during normal service hours compared to emergency hours, however, this difference did not reach statistical significance. The underlying reasons for this observation cannot be fully explained, yet it can be assumed that a greater number of staff members, who are adequately trained and confident in operating the device, are present during normal service hours. This factor may have influenced the study’s findings, as the availability of medical personnel may influence device usage, and therefore on which dogs the device was used.

The use of a CS did result in similar or higher HCT median values postoperatively compared to the dogs receiving allotransfusion or no transfusion respectively, speaking for the effectiveness of the use of a CS device regarding RBC supply. The percentage increase in HCT in the CS-group, although not significant, was the highest of the four groups, further suggesting that autologous blood has comparable effectiveness in stabilizing HCT. The autologous blood alone also reliably caused a decline in lactate over time, speaking for a reliable resuscitation of perfusion in the treated patients (35).

Dogs in the AA-, AO-, and CS-groups received plasma either pre- or post-operatively. The frequency of plasma administration between the two groups (with or without autologous blood) was not significantly different. Additionally, some dogs received plasma transfusions before surgery as part of their initial stabilization. Therefore, it is not possible to determine whether the use of the CS and autologous transfusion had an impact on coagulation or on the amount of plasma required.

Although seven dogs from both the CS-group and the-AA group received a blood volume that fell within massive transfusion limits, these dogs showed no clinically significant decrease in ionized calcium or magnesium (36). This can be explained by the fact that the autologous blood was not anticoagulated with citrate, and therefore there was no risk of citrate toxicity from the autologous blood transfusion. Autologous blood transfusion with a CS device is often anticoagulated (e.g., heparin or citrate anticoagulation) (2, 37). However, for effusions that are in contact with the pleura or peritoneum for longer than one hour, it is also possible to process the blood without anticoagulants due to the defibrination that takes place (37). At the author’s clinic, it is standard practice to give autotransfusion collected from hemorrhagic abdominal effusion using a CS device without additional anticoagulation. As a referral clinic, a time span of more than one hour between the start of hemorrhage and the start of surgery can be assumed, and the built-in filter in the CS reservoir can further minimize the risks of administering clots (38).

The evaluation of potential transfusion reactions was only possible to a limited extent due to the retrospective nature of the study. One dog experienced cardiac arrest six hours following surgery. Post-mortem diagnostic procedures were declined by the owner, precluding determination of the definitive cause of death. Furthermore, no specific risk factors for cardiac arrest (e.g., tachycardia, cardiac rhythm disturbances) were identified in the dog’s medical records prior to its arrest. As such, while the exact cause remains undetermined, it cannot be excluded that the cardiac arrest may have been a complication associated with the transfusion administered. Other causes such as thrombotic complications following splenectomy, including portal system thrombosis and pulmonary thromboembolism, secondary to hypercoagulability after splenectomy are also possible (39). Of the remaining dogs, the evaluated medical records, including anesthesia protocols and results of postoperative general physical examinations and laboratory diagnostic examinations did not reveal any severe transfusion reactions. Therefore, while our results do not allow for a definitive conclusion regarding the safety of the CS device and autologous blood, no significant increase in the risk of transfusion reactions was observed in the reported 18 cases treated with the CS device.

The significantly shorter survival time of dogs with malignant disease (100% HSA) compared to the benign causes of hemoperitoneum seems reasonable and appears similarly to previously reported publications (40, 41). No difference was found between the blood transfusion subgroups within the respective dignity groups. However, interpretation of survival times within the blood transfusion subgroups is impaired due to the not completely even distribution of malignant and benign cases. Importantly, the application of the CS device with a subsequent transfusion of the processed autologous blood product did not result in clearly correlated major or catastrophic complications and did not increase the mortality rate compared to traditional allogenic blood product application speaking for its apparent safety in the use of hemoperitoneum of splenic origin with benign and malignant dignity in the cases described in this study.

Limitations of this study include its retrospective nature, the low number of cases, the unequal and not randomized distribution along the groups including the different dignity of the pathologies and partly missing data. Regarding the evaluation of the laboratory parameters, it must be mentioned that in this study, the blood tests were not performed in a standardized manner or at standardized time points and therefore the measurement results cannot be compared unreservedly between patients. In addition, some data was missing since not all examinations were performed in all dogs. In connection with the fact that the volume of crystalloid fluids administered during volume resuscitation could not be analyzed due to lack of data, the assessment of HCT progression is mostly only an approximate. Transfusion complications could not be analyzed satisfactorily as there was a large amount of missing, undocumented data on these parameters (e.g., temperature, blood pressure, respiratory rate during and after transfusion). The stated quantification of the amount of free fluid can also only be viewed with caution, as this is the subjective assessment of the clinician performing the procedure and no standardized scoring system was used. Due to the multiple analyses between the same groups, there is an increased risk of a type 1 error. (false positive) (42). To minimize this, Dunn’s test with Bonferroni correction was chosen as the *post hoc* test. Taking all these limiting factors into account, most findings should be interpreted cautiously and only as trends. The study can be considered as a basis for further prospective and randomized studies regarding the effectiveness and usefulness of a CS device and autologous blood transfusion, but the clinical safety of CS collected autologous blood can be accepted.

In conclusion, in the present study the use of autologous blood transfusions obtained by CS device in dogs suffering from acute hemoperitoneum caused by a benign or malignant splenic disorder appeared safe and effective in the described cases. And therefore may emphasize its further application as an adjunct or alternative to traditional allogenic blood transfusions.

## Author’s note

The study results were presented at the 20th EVECC Annual Congress, June 1–3, 2023, Porto, Portugal.

## Data Availability

The data analyzed in this study is subject to the following licenses/restrictions: data of the clinic’s internal patient data software. Requests to access these datasets should be directed to blunschi.fabienne@gmail.com.
